# Effects of Hard and Soft Scalding on Defeathering and Carcass Quality of Different Breeds of Chickens

**DOI:** 10.3390/ani12223145

**Published:** 2022-11-14

**Authors:** Chia-Cheng Shung, Kun-Yi Hsin, Fa-Jui Tan, Shuen-Ei Chen

**Affiliations:** 1 Department of Animal Science, National Chung Hsing University, Taichung 40227, Taiwan; 2 The iEGG and Animal Biotechnology Center, National Chung Hsing University, Taichung 40227, Taiwan; 3 i-Center for Advanced Science and Technology (iCAST), National Chung Hsing University, Taichung 40227, Taiwan; 4 Innovation and Development Center of Sustainable Agriculture (IDCSA), National Chung Hsing University, Taichung 40227, Taiwan

**Keywords:** scalding, defeathering, broilers, country chickens, carcass quality

## Abstract

**Simple Summary:**

Soft scalding at 57 °C for 120 s is suitable for broiler chickens for desirable defeathering effects and favorable breast meat and skin color. Hard scalding at 60 °C for 60 s is optimal for layers and the local strain of RF country chickens because of its capacity to ease defeathering and retain acceptable carcass skin color and elasticity.

**Abstract:**

Current soft scalding in broilers is not applicable to various strains of chickens with different market weights and ages. This study aimed to examine the effectiveness of soft (57 °C for 120 s) and hard (60 °C for 60 s) scalding on defeathering and carcass quality of the local strain of Red Feather (RF) country chickens and determine age, breed, and body weight factor in accounting for the defeathering effectiveness using adult layers and juvenile broilers as a reference. Results showed no differences between soft and hard scalding in broilers with 60% and 80% of acceptable defeathering scores, respectively, while a significantly better effect by hard scalding was observed in adult layers, young and old RF chickens with more than 70% of birds exhibiting desirable scores and less than 20% by soft scalding. In contrast to soft scalding, hard scalding had no significant effects on breast meat color but impaired skin color in broilers as assessed by L*, a*, and b* value analysis. Interestingly, hard scalding increased breast skin yellowness and lightness in young RF chickens and lightness in old RF chickens, while there were no significant changes in layers. However, hard scalding decreased skin elasticity in layers and old RF chickens. The effectiveness of defeathering by scalding method was governed by the age to reach a certain threshold in the development of dermal architecture and feather richness, irrelevant to body weight. The alteration of carcass color primarily depends on age, body weight/breed, and their interaction. In conclusion, current soft scalding is suitable for broiler chickens for desirable defeathering effect and carcass color, whereas layers and RF chickens scaled at 60 °C for 60 s showed a better defeathering effect without unfavorable changes of breast meat and skin color.

## 1. Introduction

Chickens sent to commercial slaughter facilities are typically rendered unconscious in an electrical water bath, thereafter bled, scalded, plucked for defeathering, and eventually eviscerated. At the scalding step, carcasses are submerged in a hot water tank for a controlled duration and temperature to loosen the skin and facilitate the plucking process by rotating carcasses in the feather-picking machine, which generally can achieve 80% of defeathering [1]. Since a considerable part of chickens in the market are sold as a whole carcass, carcass appearance, particularly skin color, governs the carcass qualities that determine the suitable method of scalding. This implies that, although care must be exercised in the selection of scalding parameters that facilitate effective plucking, this step must have no perceivable defects on the carcass quality [2]. The success of the scalding procedure depends on the submersion duration and water temperature, and the combination of these two parameters greatly affects subsequent processing of the carcasses and product quality [1]. In practice, the two parameters are adjusted according to the following considerations, effectiveness of defeathering and plucking, age and weight of the chickens, carcass freezing method, and desired appearance of the final products [1,2].

The scalding process for the defeathering of chickens in a commercial slaughterhouse can be distinguished between hard and soft scalding, depending on the scalding duration and temperature. Hot scalding is typically conducted at 60–66 °C for 45–90 s. By contrast, soft scalding is usually performed with lower temperatures but a longer duration such as either 54–58 °C for 60–120 s [3], or when a lower temperature of 51–54 °C is used, the required submersion duration is further extended to 120–210 s [4,5]. For further discrimination, the scalding conditions at 54–58 °C for 60–120 s are occasionally termed medium/sub-scalding [3,6].

The soft scalding method is commonly adopted in the broiler industry for desirable defeathering effects and a favorable carcass appearance [1]. In the United States, however, most of the commercial broiler slaughterhouses use hard scalding due to the roughened skin surface for coating with batter and breading in fast food processing [7,8]. Past studies suggested that the hard and soft scalding process had no significant differences on the meat quality of broiler carcasses including color, moisture, and texture, but meat protein solubility and composition were indeed differentially affected nevertheless [9]. In case of some local strains of country chickens, however, the parameters used in broilers apparently are not applicable to various breeds of chickens with different ages and market body weights. Moreover, the intrinsic difference in the dermal biology of the chicken breed per se may govern the selection of scalding process and parameters. Red Feather (RF) chickens, a major strain of commercial country chickens in Taiwan, account for 20% of chicken meat production annually due to their intense meat flavor and chewable texture, despite that, the RF chickens were found to have a much slower growth rate (2.6–2.9 kg market weight/bird at age of 12 weeks) as compared to the commercial broilers (2.0 kg in 5 weeks) [10]. However, there are still no official recommendations and regulations by the government for the operation of the commercial slaughter of RF country chickens. Optimal parameters thus are required to standardize the scalding process of the local strain country chickens to facilitate the automatic operation of commercial electric slaughtering. In the present study, therefore, we first compared the defeathering effectiveness by the typical soft and hard scalding process and then the factors including breed, age, and body weight (BW) in accounting for the defeathering effectiveness were further examined in adolescent and adult RF country chickens using juvenile broilers and adult layers as a reference. Breast meat and skin color, and skin elasticity were also evaluated for carcass quality change.

## 2. Materials and Methods

### 2.1. Animals

To exclude the sex effect, only female chickens were selected for the study. Adolescent (12-weeks-old) female Red Feather country chickens (young RF) at market weight were purchased from a commercial farm. A flock of adult Red Feather female breeders (40-weeks-old; old RF) from the Livestock Experimental Farm, National Chung Hsing University (Taichung, Taiwan) was used and Ross 308 broiler females (Broilers) were raised to juvenile age (7-weeks-old) to achieve BW comparable to young RF chickens. Adult Hyline Brown layer females (Layers) at age of 53 weeks maintained in the Livestock Experimental Farm were used as a reference of comparable age with old RF chickens.

Birds were fasted for overnight and shipped to the slaughterhouse 2 h before the experiments. Birds were then slaughtered according to the standard chicken slaughtering process; that is, electrical stunning, bleeding, scalding, defeathering, evisceration, chilling, and refrigeration. The slaughter process was conducted in the poultry slaughterhouse located beside the Livestock Experimental Farm. 

### 2.2. Sample Preparations

Totally 160 birds were used in the study; namely 20 birds for each processing group (Broilers, Layers, young, or old RF for soft or hard scalding). The soft-scalding process was performed with birds submerged and scalded at 57 °C for 120 s before being mechanically plucked for 60 s, while in the hard-scalding process, birds were submerged and scalded at 60 °C for 60 s before being plucked for 60 s. The processing was conducted in a semi-automatic slaughter line. The sequence of slaughtering was conducted as broilers, layers, young RF, and finally old RF chickens. Chickens were first weighed, and hung upside down in the moving transport line. Subsequently, chickens were moved through an electrical bath one at a time and stunned. The electrical stunning was set as DC (direct current), 50 V, 20 mA, for 12 s. Birds were slaughtered manually by skillful workers through administration of a cut to the throat severing the carotid and jugular veins. Birds were bled for 120 s, released from the shackle, submerged in the tank of scalder, and then manually moved into the feather plucker tank. The scalder featured automatic temperature and time control, a steam circulating heating system, repeated 360° rotation submersion. After scalding, carcasses were transferred into the plucker and then plucked by mechanical rotating for 60 s. The scalder had a 7-chicken capacity for scalding and defeathering, and 4 birds per round were processed simultaneously.

Birds of each breed were divided into hard- or soft-scalding groups to assess their responses to scalding temperature and duration in terms of defeathering effectiveness and carcass qualities including breast meat color, skin color, and skin elasticity. Following evisceration of the chickens, a piece of skin 6 × 4 cm in size was removed from both sides of the sternum of each carcass, sealed in a re-sealable bag, and deposited in cold storage (4 °C) for 24 h. The skinned breast meat samples were used for immediate assessment of color change within 2 h after slaughter (defeathering and evisceration).

### 2.3. Defeathering Effectiveness

For scalding effectiveness, the parameters developed previously were adopted [2]. After plucking for 60 s, carcasses were evaluated for the effectiveness of defeathering process by two trained workers and 5 major parts of the carcass including the neck, body, wings, legs, and tail, were evaluated. A 3-level system was adopted, with level 1 indicating no residual contour or flight feathers, while level 2 with 1 to 3, and level 3 with more than 3 contour or flight feathers that remained in each of the major part [11].

### 2.4. Skin and Breast Meat Color Analyses

The color of breast meat at the site of skinning was measured within 2 h after evisceration. The breast skin samples were removed from cold storage, allowed to return to room temperature for 1 h, and then spread evenly on an opaque white board for color measurement. Breast samples from both sides of each bird were used for color measurement. Measurement was made at the top of eminence site in the anterior part of breast sample and 1 cm below or above the top with 3 replicates in the reading.

Color analyses were conducted using a handy colorimeter (NR-3000, Nippon Denshoku, Tokyo, Japan); three parameters, L* (lightness), a* (redness), and b* (yellowness) were recorded. Before use, the handy colorimeter was calibrated by measuring the color of a white correcting plate (X = 85.81, Y = 90.15, Z = 96.16). The L* (lightness), a* (redness), and b* (yellowness) values (i.e., the CIELAB color space established by the International Commission on Illumination) of the samples were measured [12].

### 2.5. Skin Elasticity Test Elasticity

After skin color measurement, the skin samples were excised into stripes with 3 ± 0.5 cm in length and 0.5 ± 0.1 cm in width and the connective tissue and flesh that remained underneath the skin was removed using a surgical knife. A shear tester (G-R Manufacturing Co., Manhattan, KS, USA) with the Warner-Bratzler shear force up to 25 kg was then employed to determine the magnitude of tensile force [13]. The skin strip was hung over the bottom of the dull blade and pulled upwards to pass the cleft of the hold frame. During the passage through the cleft, the strip was stretched gradually to a breaking point and thus allowed tensile force reading. Each skin sample was tested twice.

### 2.6. Statistics

Data of body weight were analyzed by one-way ANOVA using breed as the variable. A two-way analysis of variance (ANOVA) using scalding condition (hard vs. soft) × age (7-weeks old broilers vs. other breeds of birds at older ages) or scalding condition (hard vs. soft) × BW (layers with 1.8 kg/bird vs. other breeds of birds at 2.6–2.7 kg/bird) was performed to analyze the main effect. If an interaction existed between scalding and age or between scalding and body weight, a Student’s *t*-test or Duncan’s multiple-range test (SAS 9.4) was used for means comparisons. The defeathering scoring results were re-grouped for effective defeathering with the total scores less than 9 for an acceptable defeathering procedure and larger than 10 for ineffectiveness. All factor effects on scalding effectiveness were calculated with the Chi-square likelihood ratio test and analyzed by Nominal Logistic Regression. Data were presented as mean and comparison results with *p* < 0.05 level were regarded as a significant difference.

## 3. Results

### 3.1. Defeathering Effectiveness

For comparable variables, namely age and body weight, juvenile broilers, adult layers, and old RF country chickens with optimal ages and body weights were used in the study. Broiler were raised to age of 7 weeks to achieve a similar body weight (2.6 kg in average) comparable to the commercial RF country chickens at market weight (2.7 kg at age of 12 weeks) and a flock of 40-weeks-old RF female breeders (2.6 kg in average) was used as a comparable counterpart with the same breed and body weight but different ages. Layers with significantly lower body weight (1.8 kg in average, *p* < 0.05, Table 1) at age of 53 weeks comparable to the age of old RF chickens were tested for body weight effect.

**Figure 1:**
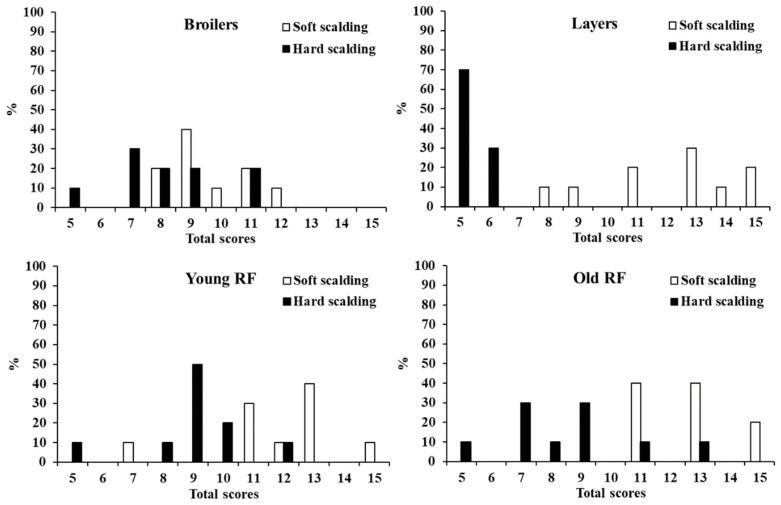
The distribution of total defeathering score of different breeds of chickens. After soft (57 °C/120 s) or hard (60 °C/60 s) scalding, carcasses were automatically plucked for 60 s in the tank. Five major parts of the carcass including neck, body, wings, legs, and tail were evaluated for defeathering effects based on the 3-level system. The scores of each part of individual bird were combined to produce a total with a range from a minimal score 5 to 15 for maximum. Results were expressed as a percentage of total birds of each breed in the 5–15 scale (n = 20).

The effectiveness of soft and hard scalding on defeathering was judged at the five major parts including the neck, body, wings, legs, and tail, using the 3-level system. Since the effectiveness of defeathering is evaluated in a whole carcass, the scores of each part of individual bird were combined to produce a total score ranging from minimal 5 to 15 for maximum. The distribution of defeathering score showed a two-peak mode with a distinctive nadir cut-off at score 10 in the 4 breeds of chickens (Figure 1), in which the defeathering scores of broilers by soft and hard scalding were distributed mainly in the scale less than 9, while in layers and young and old RF chickens, hard and soft scalding caused a major distribution within the score less than 9, and larger than 10, respectively. 

**Figure 2:**
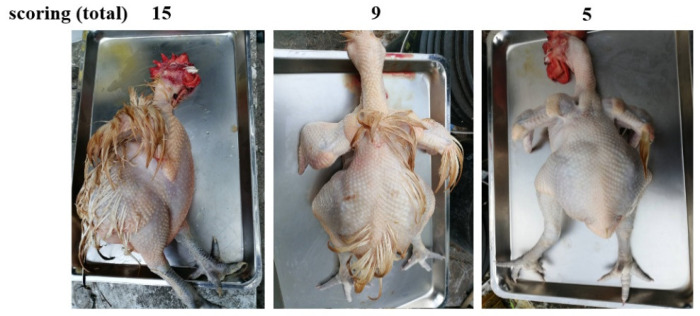
The typical carcass morphology of defeathering scores of young Red Feather country chickens. The scores of each part (neck, body, wings, legs, and tail) of individual bird were combined to produce a total with a range from a minimal score 5 to 15 for maximum. A typical carcass morphology with total scores of 15, 9, or 5 is shown.

**Figure 3:**
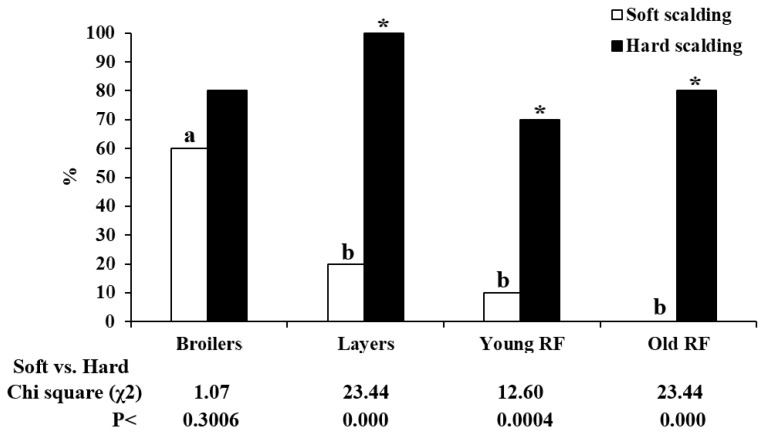
The defeathering effectiveness of soft and hard scalding in different breeds of chickens. According to the distribution of defeathering scores (Figure 1), the total scores less than 9 were regarded as effective defeathering procedure, while scores larger than 10 were unacceptable. Results were expressed as a percentage of total birds of each group with total score less than 9 in the 5–15 scale. Means with different superscript letters within the same row differ significantly among breeds (*p* < 0.05, n = 20). * significant differences vs. soft scalding (57° C/120 s) (*p* < 0.05, n = 20). Broilers (2.6 kg/bird) and layers (1.8 kg/bird) at age of 7 and 53 weeks, respectively. RF: Red Feather country chickens at age of 12 (young, 2.7 kg/bird) and 40 (old, 2.6 kg/bird) weeks.

Based on the distribution, the scoring results were re-grouped for net effectiveness with the scoring less than 9 for acceptable defeathering procedure and larger than 10 for ineffectiveness (Figure 2). Results of re-grouping showed that both soft and hard scalding in broilers achieved a similar effectiveness with 60% and 80% of birds graded with acceptable defeathering scores, while layers, in young and old RF chickens showed a significantly improved defeathering effect by hard scalding with 100%, 70%, and 80% of birds achieving acceptable scores, respectively, and less than 20% by soft scalding (Figure 3, *p* < 0.05). These results clearly concluded hard scalding for effective defeathering in layers, in young and old RF chickens. Under soft scalding conditions, juvenile broiler chickens showed a significantly higher defeathering effectiveness than adolescent and adult RF chickens with comparable body weight, as well as than adult layers despite much lighter body weight (Figure 3, *p* < 0.05), while no differences by hard scalding were found among the four breeds of chickens. Accordingly, the age to reach a certain threshold in the development of dermal architecture and feather richness rather than body weight predominates in determining the effectiveness of the defeathering process.

### 3.2. Breast Muscle Color

Results by ANOVA using scalding condition (hard vs. soft) × age (7-weeks old broilers vs. other breeds of birds at older ages) as variables suggested no significant differences by the main effects in breast meat color, nor by their interaction (Table 2). A significant effect on b* values (yellowness) by BW (*p* < 0.026) was observed when assessed by scalding condition (hard vs. soft) × BW (layers with 1.8 kg/bird vs. other breeds of birds at 2.6–2.7 kg/bird) ANOVA (Table 2). However, no significant differences were observed among the breeds of chickens under soft or hard scalding process (*p* > 0.05).

A very close to significant level in L* values (lightness) by age (*p* < 0.056), and L* and a* (redness) values by scalding condition (*p* < 0.083 and 0.054) were found in scalding × age and scalding × BW analysis, respectively. Since young, old RF chickens, and broilers had comparable body weight but different ages, while varied in body weight with adult layers, this susceptibility of breast meat color change to hard scalding (vs. soft scalding) thus is attributed to the age and breed, by which younger birds may have loosened muscle structure and thus are sensitive to protein denaturation, and hemochrome oxidation leading to color change [1,7]. Moreover, heavier BW of broilers and RF chickens, which reflects breed differences for higher proportions of muscle mass in meat-type chickens, may also contribute to the intrinsic difference in muscle pigment and hemochrome content leading to differential resistances to scalding conditions.

### 3.3. Post Defeathered Skin Color

Age and BW exerted a significant effect on L* and/or a* values in breast skin color (*p* < 0.001, Table 3), while the interaction of scalding × age also significantly affected L* and a* values (*p* < 0.015 and 0.013, respectively). Scalding condition had a very close to significant effect on a* and b* values (*p* < 0.095 and 0.054) in scalding × BW and scalding × age analysis, respectively (Table 3).

Under soft scalding, a* values were ranked as broilers > young = old RF chickens > layers, L* value as layers > broilers > young = old RF chickens, while b* value as broilers = layers > young RF chickens (*p* < 0.05) with no differences to old RF chickens (Table 3). Under hard scalding, a* value was ranked as broilers > young > old RF chickens > layers, L* values as layers > old RF chickens > broilers = young RF chickens, and b* value as layers = young RF > broilers (*p* < 0.05) with no differences to old RF chickens (Table 3). 

In contrast to soft scalding, hard scalding decreased L* and b* values in broilers but promoted L*, and b* values in young RF chickens and b* values in old RF chickens with comparable body weight (*p* < 0.05, Table 3), while they had no significant effects on adult layers despite lighter body weight. These differential results suggested that age and BW due to breed differences, and their interaction govern the factors in determining skin color alteration by hard scalding. The changes in L* value under hard scalding likely reflect liquefied follicles and subcutaneous fat by higher temperatures, thus causing the skin to become brighter in appearance [1,3]. Likewise, increased temperatures may cause the corneum and flavones on the stratum skin to detach easily, thus lowering the b* values [1]. The alteration of skin color depends on age, breed, and their interaction regardless of body weight.

### 3.4. Tensile Test for Scalded Skin

Scalding condition (*p* < 0.027 and 0.044) and age (*p* < 0.001) significantly affected breast skin tensile strength (Table 4). Under soft scalding, the tensile strength was ranked as old RF chickens > layers = young RF chickens > broilers, while under hard scalding, the rank was old RF chickens > young RF chickens > broilers (*p* < 0.05, Table 4) and layers had higher shear force than broilers (*p* < 0.05) but no differences with old and young RF chickens (Table 4). In contrast to soft scalding, a significant decrease in breast skin tensile strength by hard scalding was observed in broilers, layers, and old RF chickens, but not in young RF chickens. These results suggest that adolescent birds’ skin with organized histological structures at the dermal-epidermal junction, such as complex elastic fiber arrays and basket weave fibril collagen has tougher resistance to physical deformation [14,15].

## 4. Discussion

The present results manifested that the current soft scalding method at 57 °C for 120 s is suitable for broiler chickens for desirable defeathering effects and favorable carcass appearance, while layers and juvenile and adult RF country chickens by hard scalding at 60 °C for 60 s exhibited better defeathering without significant unfavorable changes on breast meat and skin color. The age to reach a certain threshold rather than body weight was concluded to govern the effectiveness of defeathering by scalding process, while the age, BW/breed, and their interaction are the causative factors contributing to the resistance of carcass color alterations. The certain threshold can be attributed to the development of dermal architecture and feather richness [1,14,15].

Selection of scalding parameters depends on factors such as the effectiveness of feather removal, age of the birds, chilling method to follow, and desired appearance of processed carcasses and final products [1]. The combination of temperature and duration parameter of scalding is widely used within today’s poultry industry. Since broilers are rather immature and have delicate skin, soft flesh, and weak bones, an increased plucking time is not recommended due to the risk of bruises and bone fractures. Suitably extending the submersion duration at a lower scalding temperature to improve defeathering effectiveness is advisable [3,4]. Soft scalding at 57 °C for 120 s is commonly used by current slaughterhouses in the broiler industry [1]. This process can retain the outer yellow layer of the skin surface of broiler and turkey carcasses for favorable appearances. The current soft scalding has no negative effects on the outer skin layer maintaining the waxy and yellow pigments intact on the surface, but its defeathering effectiveness is much lower in turkeys than that performed in broilers [1,3,4]. Accordingly, factors such as body weight, age, and species in the contribution to defeathering effectiveness in turkeys require further confirmation. An insufficient scalding process can increase the difficulty of plucking and thereby the presence of too many residual feathers would impede the automatic slaughtering process and increase the need of manual labor.

Hard scalding, namely, at higher temperatures in a shorter duration such as 60 °C for 60 s, is more effective to loosen feathers from follicles and is commonly used for waterfowls, turkeys, and spent layer hens. The higher temperatures of hard scalding cause the outer layer of skin epidermis more loosen and thus is easily removed later during the feather picking operation leading to a white carcass [1]. In the present study, soft scalding was insufficient for the defeathering in layers and RF chickens, possibly because these chickens are in adolescent or adult ages and thus have higher feather richness and tougher architectures in the dermal layer, subcutaneous connective tissues, and hair follicles to hold feathers [1]. Because of the risk of visible external damages on the carcasses, an increased time of mechanical rotating for feather plucking is also not recommended. The external damages of carcass appearances by increased plucking time may be exacerbated in the RF country chickens with thinner dermal layer due to their heredity [10]. Instead, optimization of scalding temperature and duration is advised for effective defeathering. Specifically, a scalding process for older chickens was suggested to be 60–63 °C or maintained at 60 °C, and the scalding duration can be prolonged up to 90 s [16]. Over-scalding due to higher temperatures can result in a banding appearance and liquefied fat on the surface of breast meat and skin [1].

Color is one of the major quality attributes of poultry carcasses and is directly associated with the properties of the meat varying in breeds, ages, feed, and management. Skin color of the carcasses attracts the first sight of consumers and governs their choice of the products [17]. The pigmentation of chicken skin is influenced by multiple factors, including heredity, age, the pigments in the diet [18], health condition [19], and the slaughtering process [1,3,7]. The present results suggested that sex maturity is the causative factor to contribute to breast meat color change by hard scalding, by which immature birds may have loosened muscle structures and thus are more susceptible in protein denaturation and hemochrome and pigment oxidation. A high scalding temperature results in the color change in chicken breast meat, particularly, the ventral part, affecting the external lightness of the meat [20]. An increase in scalding temperatures from 54 to 58 °C caused dramatic color changes and protein denaturation leading to lighter color in chicken breast meat within 24 h after slaughter, a transformation in appearance that is sufficient to affect consumers’ purchase intension [21]. Despite the reports, some studies concluded that hard scalding gives rise no significant effects on breast meat color [12,22,23]. This contradiction may be attributed to the durations and simultaneous bird numbers in the scalder tank, the timing of assessment for breast meat color change after slaughter process, as well as the duration after refrigeration [20,24,25]. Nevertheless, an adequate combination of scalding temperature and duration was advised in previous studies to prevent unfavorable changes in chicken meat quality [20,21,24].

Past studies suggested that increased scalding temperatures can remove the corneum and cuticle in the breast skin and decrease its b* values leading to a pale and less-yellow appearance of the carcass [7]. Soft scalding can loosen feathers without damaging the skin and thereby retain the stratum corneum and flavones leading to a lighter and yellowish appearance in the skin [1]. Therefore, soft scalding is widely used in the broiler industry to minimize color change of the breast meat. Hard scalding can effectively loosen up follicles and lead to skin appearing slightly pale. Under the air-cooling process, however, the skin of carcass tends to be dehydrated, and thus hard scalding may exacerbate the change in carcass color turning a darker, yellower color, when compared with water cooling or vapor cooling [3,25]. Furthermore, most of the local country chickens have a stronger and reddish muscle color due to older ages at the market weight, as well as thinner skin due to heredity [10]. This stronger hue underneath the dermal layers therefore can darken the superficial skin color rendering an unfavorable appearance of the carcass when sold as a whole. 

Broiler carcasses are typically dissected for sell, and breast meat is among the most popular on the market. Different from broilers, local country chickens with deeper skin color are regarded as portraying meat from healthy and high-quality birds in Asian countries and mostly are sold as a whole carcass. This results in a particular attention for carcass skin color change by scalding procedure. Moreover, the meat of spent layers is commonly used for processed products such as meat floss and meat balls. Accordingly, the parameters of slaughter procedure in commercial electric slaughter houses should be balanced according to chicken age and breeds to ensure the effectiveness of defeathering and carcass quality. The present study demonstrated hard scalding at 60 °C for 60 s for effective defeathering in layers and RF country chickens without unfavorable changes of breast meat and skin color.

## 5. Conclusions

The current scalding procedure at 57 °C for 120 s in broiler chickens can achieve a desirable defeathering effect and favorable carcass color. Hard scalding at 60 °C for 60 s had effective defeathering for layers and the local strain of RF country chickens with acceptable carcass breast and skin color. The defeathering effectiveness by scalding depends on the age to reach a certain threshold in the development of dermal architecture and feather richness, while carcass color change is governed by age, BW/breed, and their interaction.

## Figures and Tables

**Table 1:** Age and body weight of chickens.

Breed	Age (Weeks)	Body Weight (g/Bird)
Broilers	7	2625 ± 324 ^a^
Layers	53	1763 ± 109 ^b^
Young RF	12	2676 ± 205 ^a^
Old RF	40	2579 ± 335 ^a^

Means (±SD) with different superscript letters differ significantly (*p* < 0.05, n = 20). RF: Red Feather country chickens at age of 12 (young) and 40 (old) weeks.

**Table 2:** Effects of soft and hard scalding on breast meat color of different breeds of chickens.

CIELAB Color Space	Soft Scalding (57 °C/120 s)				Hard Scaling (60 °C/60 s)				SEM
	Broilers	Layers	Young RF	Old RF	Broilers	Layers	Young RF	Old RF	
L*	56.85	54.41	46.73	57.13	59.41	59.33	50.84	60.17	0.76
a*	5.43	7.15	10.86	5.61	4.94	4.42	8.91	3.16	0.45
b*	4.22	4.08	4.78	5.52	6.33	3.95	6.93	7.71	0.37
Source of variation	*p*-value ^1^								
	Main Effect		Interaction		Main Effect		Interaction		
	Scalding	Age	Scalding × Age		Scalding	BW	Scalding × BW		
L*	0.192	0.056	0.871		0.083	0.339	0.291		
a*	0.355	0.142	0.159		0.054	0.498	0.491		
b*	0.228	0.799	0.214		0.727	0.026	0.612		

^1^ A two-way analysis of variance (ANOVA) using scalding (hard vs. soft) × age (7-weeks old broilers vs. other breeds of birds at older ages) or scalding (hard vs. soft) × body weight (layers with 1.8 kg BW vs. other breeds of birds at 2.6–2.7 kg) was performed to analyze the main effect. L* = lightness, a* = redness, b* = yellowness. Broilers (2.6 kg/bird) and layers (1.8 kg/bird) at age of 7 and 53 weeks, respectively. RF: Red Feather country chickens at age of 12 (young, 2.7 kg/bird) and 40 (old, 2.6 kg/bird) weeks.

**Table 3:** Effects of soft and hard scalding on breast skin color of different breeds of chickens.

CIELAB Color Space	Soft Scalding (57 °C/120 s)				Hard Scaling (60 °C/60 s)				SEM
	Broilers	Layers	Young RF	Old RF	Broilers	Layers	Young RF	Old RF	
L*	71.08 ^b^	73.47 ^a^	66.24 ^c^	66.68 ^c^	69.28 ^c^ *	74.12 ^a^	68.29 ^c^ *	72.07 ^b^*	0.41
a*	17.13 ^a^	3.26 ^c^	11.29 ^b^	11.77 ^b^	19.71 ^a^	3.52 ^d^	14.51 ^b^	12.12 ^c^	0.71
b*	17.33 ^a^	16.51 ^a^	15.25 ^b^	16.83 ^ab^	15.64 ^b^ *	16.74 ^a^	16.55 ^a^ *	15.95 ^ab^	0.17
Source of variation	*p*-value ^1^								
	Main Effect		Interaction		Main Effect		Interaction		
	Scalding	Age	Scalding × Age		Scalding	BW	Scalding × BW		
L*	0.619	0.947	0.015		0.095	0.001	0.415		
a*	0.339	0.001	0.406		0.167	0.001	0.188		
b*	0.054	0.634	0.013		0.805	0.349	0.400		

^1^ A two-way analysis of variance (ANOVA) using scalding (hard vs. soft) × age (7-weeks old broilers vs. other breeds of birds at older ages) or scalding (hard vs. soft) × body weight (layers with 1.8 kg BW vs. other breeds of birds at 2.6–2.7 kg) was performed to analyze the main effect. * significant difference vs. soft scalding (57° C/120 s) within the same measurement and breed (*p* < 0.05, n = 20). Means with different superscript letters within the same row differ significantly among breeds (*p* < 0.05, n = 20). L* = lightness, a* = redness, b* = yellowness. Broilers (2.6 kg/bird) and layers (1.8 kg/bird) at age of 7 and 53 weeks, respectively. RF: Red Feather country chickens at age of 12 (young, 2.7 kg/bird) and 40 (old, 2.6 kg/bird) weeks.

**Table 4:** Effects of soft and hard scalding on skin tensile force of different breeds of chickens.

	Soft Scalding (57 °C/120 s)				Hard Scaling (60 °C/60 s)				SEM
	Broilers	Layers	Young RF	Old RF	Broilers	Layers	Young RF	Old RF	
Force (kg)	4.57 ^c^	8.08 ^b^	6.35 ^b^	12.3 ^a^	3.85 ^c^ *	6.93 ^ab^ *	5.70 ^b^	7.39 ^a^ *	0.33
Source of variation	*p*-value ^1^								
	**Main Effect**		**Interaction**		**Main Effect**		**Interaction**		**BLANK**
	**Scalding**	**Age**	**Scalding × age**		**Scalding**	**BW**	**Scalding × BW**		
	0.027	0.001	0.260		0.044	0.763	0.434		0.434

^1^ A two-way analysis of variance (ANOVA) using scalding (hard vs. soft) × age (7-weeks old broilers vs. other breeds of birds at older ages) or scalding (hard vs. soft) × body weight (layers with 1.8 kg BW vs. other breeds of birds at 2.6–2.7 kg) was performed to analyze the main effect. * significant difference vs. soft scalding (57° C/120 s) within the same measurement and breed (*p* < 0.05, n = 20). Means with different superscript letters within the same row differ significantly among breeds (*p* < 0.05, n = 20). L* = lightness, a* = redness, b* = yellowness. Broilers (2.6 kg/bird) and layers (1.8 kg/bird) at age of 7 and 53 weeks, respectively. RF: Red Feather country chickens at age of 12 (young, 2.7 kg/bird) and 40 (old, 2.6 kg/bird) weeks.

## Data Availability

The data presented in this study are available on request from the corresponding authors.
